# Unique Cytokine Signature in the Plasma of Patients with Fibromyalgia

**DOI:** 10.1155/2014/938576

**Published:** 2014-03-11

**Authors:** Jamie Sturgill, Elizabeth McGee, Victoria Menzies

**Affiliations:** ^1^School of Nursing, Virginia Commonwealth University, Richmond, VA 23298, USA; ^2^Institute of Women's Health, Virginia Commonwealth University, Richmond, VA 23298, USA; ^3^Department of Obstetrics, Gynecology and Reproductive Sciences, University of Vermont, Burlington, VT 05401, USA

## Abstract

Fibromyalgia (FMS) is a chronic pain syndrome with a complex but poorly understood pathogenesis affecting approximately 10 million adults in the United States. The lack of a clear etiology of FMS has limited the effective diagnosis and treatment of this debilitating condition. The objective of this secondary data analysis was to examine plasma cytokine levels in women with FMS using the Bio-Plex Human Cytokine 17-plex Assay. Post hoc analysis of plasma cytokine levels was performed to evaluate patterns that were not specified *a priori*. Upon examination, patients with FMS exhibited a marked reduction in T_H_2 cytokines such as IL-4, IL-5, and IL-13. The finding of this pattern of altered cytokine milieu not only supports the role of inflammation in FMS but also may lead to more definitive diagnostic tools for clinicians treating FMS. The T_H_2 suppression provides strong evidence of immune dysregulation in patients with FMS.

## 1. Introduction

Fibromyalgia (FMS) is a chronic pain syndrome in which pathogenesis is complex and cure is not known. It affects approximately 10 million adults in the United States with an estimated 90% of diagnoses being reported in women [1]. The symptom profile of FMS includes pain, fatigue, and distressed mood. Sequelae of FMS include physical and psychological distress, loss of work productivity, reduced quality of life, and increased use of health resources. Annual expenditures for the diagnosis and treatment of FMS are estimated at approximately $20 billion, thus presenting a significant burden to patients, their families, and society [2, 3]. Although the incidence of FMS is rising, the etiology remains unclear. A major theory is that inflammatory mediators lead to complex neuroendocrine aberrations of the hypothalamic-pituitary-adrenal (HPA) axis [4]. Altered levels of cytokines have been associated with symptoms of pain, fatigue, and distressed mood in multiple conditions including painful peripheral neuropathies, hepatitis C, cardiovascular disease, and cancer [3, 5, 6]. This symptom profile mimics the representative symptoms of FMS. Thus, it is theoretically plausible that these nonspecific inflammatory mediators may also contribute to the symptoms of pain, fatigue, and distressed mood in FMS. To date, results of studies examining the association of cytokine alterations with FMS and its symptoms have been mixed [7–9]. Although researchers have suggested FMS as being an inflammatory state related to a dysregulated immune system or altered stress response, the pathophysiological role of cytokines continues to remain unclear [9, 10]. Because there are no diagnostic markers for FMS as well as no identified etiology for the development of FMS, researchers are still searching for mechanistic signs to identify those who already have or those who are at risk for developing fibromyalgia.

T helper lymphocytes are defined by expressing the cell surface molecule known as CD4 and are subdivided further based on the cytokines that they produce. The discovery of the T_H_1 and T_H_2 paradigm [11] was a pivotal breakthrough in the field of immunology. This balance and counterbalance of inflammatory mediators were delineated and ultimately led to fundamental additions to the knowledge base of cytokine biology we understand today. Although T_H_ subsets have expanded far beyond the initial discovery to include T_H_17, T_H_9, T_FH_ and others probably yet to be discovered, we can still use the T_H_1 and T_H_2 paradigm to better understand inflammation at both the bench and bedside. T_H_1 immune responses are historically associated with antitumor and antiviral responses, whereas T_H_2 are associated with humoral immune responses. However, today these designations are being expanded to include other disease states. For example these helper T cell derived cytokines are being examined in disease states such as schizophrenia [12], depression [13], and chronic pain [14].

The purpose of the secondary data analysis in this study was to examine cytokine profiles in women in diagnosed with FMS and to determine if relationships existed among the secreted cytokines detected in the plasma and to determine if any unique cytokine patterns that emerge correlate with disease symptoms.

## 2. Patients and Methods

### 2.1. Patients

Two separate studies were conducted: one preliminary and one for validation. Both were approved by the Institutional Review Board of Virginia Commonwealth University. Our preliminary study involved 42 females, whereas the validation study was comprised of 63 females. Inclusion criteria included age ≥18, female, diagnosis of FMS as defined by the 1990 American College of Rheumatology (ACR) criteria, no known major psychiatric or neurological conditions that would interfere with study participation, and an ability to understand and sign the consent form. The 1990 ACR criteria for fibromyalgia require that an individual has both a history of chronic widespread musculoskeletal pain (more than 3 months) and the finding of 11 of 18 possible tender points upon physical examination [15]. Both studies were completed prior to the publication of the 2010 revised FMS diagnostic criteria [16]. Exclusion criteria included presence of other systemic rheumatologic conditions, being immunocompromised (e.g., diagnosis of HIV/AIDs), receiving corticosteroid treatments, being treated for cancer, and/or being pregnant. Self-reported diagnosis of FMS was confirmed by the participant's primary physician or rheumatologist.

### 2.2. Questionnaires

In both studies, study participants completed self-report form to collect data regarding age, race/ethnicity, marital status, length of time since diagnosis of FMS, socioeconomic status and psychiatric, medical and medication history. Stress was measured using the Perceived Stress Scale (PSS). The 10-item PSS measures the degree to which the individual perceived events in her life over the previous month to be stressful. The scale has an internal reliability of 0.78 and demonstrated construct validity [17]. Pain was measuredusing the Brief Pain Inventory (BPI) Short Form [18]. The BPI assesses pain severity (BPI-S) and pain interference (BPI-I) using 0–10 numeric scales for item rating; higher scores indicate increased pain/interference. Pain severity indicates the intensity of the pain experienced, while pain interference measures the degree to which pain interferes with activities of daily living. In widespread testing, the Cronbach's alpha reliability ranges from 0.71 to 0.91 [19]. Fatigue was measuredusing the Brief Fatigue Inventory (BFI), a simple, 9-item scale that taps into a single dimension of fatigue severity and the interference fatigue creates in daily life. A score of 7 or higher indicates severe fatigue [20]. The BFI has demonstrated excellent reliability in clinical trials, ranging from 0.82 to 0.97 [19]. Depression was measured using the Center for Epidemiological Studies Depression Scale (CES-D). The CES-D is a 20-item self-report instrument comprised of four factors assessing cognitive and affective components of depression. This instrument has very good construct validity, internal consistency, and test-retest reliability [21].

### 2.3. Immunological Assays

Blood samples were collected into heparinized vacutainer tubes for measuring immune markers. Blood was centrifuged for separation of plasma, and all specimens were aliquoted immediately, frozen, and stored at −80° until all samples were collected. All samples were assayed together to reduce interassay variability.

Plasma levels of cytokines such as interleukin (IL) 1beta (IL-1*β*), IL-2, IL-4, IL-5, IL-6, IL-7, IL-10, IL-12p70, IL-13, IL-17, G-CSF, GM-CSF, IFN-*γ*, and TNF as well as chemokines such as CXCL8 (IL-8), CCL2 (MCP1), and CCL4 (MIP1*β*) were analyzed using the 17-plex Bio-Rad (Bio-Rad; Hercules, CA) cytokine, chemokine, and growth factor assay kit per manufacturer's protocol.

### 2.4. Data Analysis

All data are presented as the mean +/− standard error of the mean (SEM). All secondary analysis was performed using SigmaPlot software.

## 3. Results

### 3.1. Initial Study

The patient demographics can be found in Table 1. Post hoc analysis of plasma cytokine levels was performed to determine if patterns appeared that were not specified* a priori. *Given the fact that the patients were of only two races, analysis was first performed to determine if differences existed in the cytokine levels of Caucasian women with FMS versus African American women with FMS. Using Mann-Whitney *U* tests, 16 of the 17 cytokines assayed displayed no statistical difference among race (data not shown) and were thus used for further analysis. Power analysis was performed to ensure a 95% confidence level with a confidence interval of 15%. Results from this analysis indicated that an appropriate *N* for cytokines would be equal to or greater than 21. We then eliminated three more cytokines for further analysis given that the *N* of patients with detectable levels was below 21. These stringency criteria left 13 cytokines and chemokines for further examination. The values for our FMS patients are presented in Table 2.

Due to the fact that at study initiation normal controls were not collected, we used plasma values reported in the literature [22–30]. While we acknowledge this weakness, we are confident that the analyses are still powerful and will warrant further examination into cytokine deviations present in FMS. In order to move forward in the study, we only looked at cytokines that were at least 2X greater in difference than 2SEMs of the mean. Thus, IL-4, IL-5, IL-13, and GCSF were further examined. Upon further scrutiny of the remaining 4 cytokines, a stark pattern began to emerge in that these cytokines are associated with T_H_2 immunity.

### 3.2. Validation Study

To confirm these findings, a secondary set of FMS were analyzed. These patients were recruited for a different study and thus were completely independent of the subjects enrolled in the initial study. The patient demographic can be found in Table 3. Given the drastic differences observed in the original data set for IL-4, IL-5, IL-13, and GCSF, these cytokines were further scrutinized in the independent validation data set. Using the aforementioned criteria, we confirmed that IL-4, IL-5, and IL-13 are indeed suppressed in patients with FMS (Table 4).

### 3.3. Correlation Analysis

**Figure 1:**
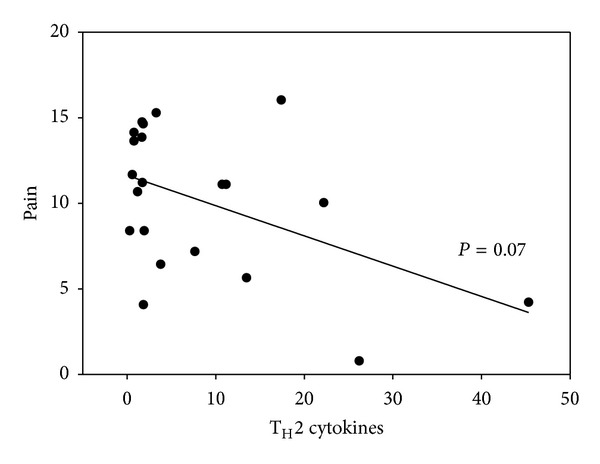
Correlation of pain and T_H_2 cytokines. A total T_H_2 value was determined by adding plasma values of IL-4, IL-5, and IL-13. A total pain value was determined adding BPI-I and BPI-S using the Brief Pain Inventory scale as described in the material and methods. A Spearman's Correlation was performed on the two values using SigmaPlot and data is shown. Correlation coefficient −0.391, *P* value 0.07.

Upon the observation that patients with FMS had suppression in T_H_2 cytokines, we began to explore the potential relationship between T_H_2 immunity and the psychometrics obtained from the individuals. While no correlations proved significant among cytokine levels and fatigue, depression, or stress (data not shown), there is a trend towards significance when we compared T_H_2 cytokine levels and pain (*P* = 0.07, Spearman's) as shown in Figure 1.

## 4. Discussion

Although not classified as an immune disease by nature, our group as well as others has reported cytokine and immune alterations in patients with FMS [3, 7, 9, 15, 23]. Given the fact that disease etiology is still uncertain, further research is needed in the field to help uncover exact disease pathology in hopes to provide better therapeutic options the millions of FMS patients worldwide. The purpose of this analysis was to examine cytokine alterations in patients with FMS that were not determined* a priori*. Comparing the observed cytokines in the plasma of these patients we noticed a stark decrease in the amount of T_H_2 cytokines produced (IL-4, IL-5, and IL-13) as those values reported by other groups in the literature. We extended our analyses to include a secondary, independent data set and once again this unique cytokine signature was observed. To examine a potential underlying cause for the T_H_2 suppression, we then correlated cytokine levels to pain, stress, fatigue, and depression which are all symptoms shared in the FMS spectrum. When pain and T_H_2 levels were compared we observed a trend that approached statistical significance. Interleukin 4, the classic T_H_2 cytokine has been shown to have both anti-inflammatory and analgesic properties in murine models of mechanical [31] as well as having lower gene expression and serum levels in patients with widespread pain syndromes [6]. IL-13 exhibits analgesic properties in a murine model of* L. major* infection [32], whereas little is reported in regard to IL-5's ability to combat pain. Thus, our preliminary findings suggest that further research into the T_H_1-T_H_2 imbalance in FMS and its implication in pain are certainly warranted.

## Figures and Tables

**Table 1:** Patient demographics.

Number of patients enrolled	*N* = 42
Female	*N* = 42 (100%)
Race	*N* = 17 African American (41.9%)
	*N* = 25 Caucasian (58.1%)
Age	Average = 48.7 (Range 26–75)

**Table 2:** Cytokine measured.

Cytokine/chemokine	Average ± SEM	Normal range
CCL2	38.0 ± 3.1	50.0–298.8
CXCL8	14.5 ± 10.8	6.4–20.4
IL-l*β*	4.3 ± 3.4	0.0–1.2
IL-4	0.6 ± 0.1	5.5–12.5
IL-5	1.2 ± 0.1	6.0–44.5
IL-6	9.1 ± 2.9	11.0–17.0
IL-7	9.5 ± 0.8	0.0–22.0
IL-12p70	5.7 ± 0.6	1.7–2.5
IL-13	2.8 ± 0.3	12.0–47.9
GCSF	89.8 ± 6.1	0.0–34.4
GMCSF	26.9 ± 5.9	2.0–48.0
IFN*γ*	32.9 ± 6.5	1.2–25.0
TNF	16.4 ± 2.1	2.1–46.0

**Table 3:** Patient demographic.

Number of patients enrolled	*N* = 63
Female	*N* = 63 (100%)
Race	*N* = 18 African American (28.6%)
	*N* = 45 Caucasian (71.4%)
Age	Average = 47 (Range 18–71)

**Table 4:** Cytokines validated.

Cytokine/chemokine	Average ± SEM
IL-4	1.8 ± 1.1
IL-5	1.0 ± 0.4
IL-13	4.4 ± 1.4
GCSF	12.4 ± 2.4
